# Avian Influenza (H5N1) Virus of Clade 2.3.2 in Domestic Poultry in India

**DOI:** 10.1371/journal.pone.0031844

**Published:** 2012-02-20

**Authors:** Shanmuga Nagarajan, Chakradhar Tosh, David K. Smith, Joseph Sriyal Malik Peiris, Harshad Vinayakrao Murugkar, Rajangam Sridevi, Manoj Kumar, Megha Katare, Rajlaxmi Jain, Zohra Syed, Padmanava Behera, Chung L. Cheung, Rekha Khandia, Sushil Tripathi, Yi Guan, Shiv Chandra Dubey

**Affiliations:** 1 High Security Animal Disease Laboratory, Indian Veterinary Research Institute, Anand Nagar, Bhopal, India; 2 State Key Laboratory for Emerging Infectious Diseases, The University of Hong Kong, Hong Kong Special Administrative Region; 3 State Key Laboratory of Emerging Infectious Diseases, Li Ka Shing Faculty of Medicine, University of Hong Kong, International Institute of Infection and Immunity, Shantou University Medical School, Shantou, People's Republic of China; Erasmus Medical Center, The Netherlands

## Abstract

South Asia has experienced regular outbreaks of H5N1 avian influenza virus since its first detection in India and Pakistan in February, 2006. Till 2009, the outbreaks in this region were due to clade 2.2 H5N1 virus. In 2010, Nepal reported the first outbreak of clade 2.3.2 virus in South Asia. In February 2011, two outbreaks of H5N1 virus were reported in the State of Tripura in India. The antigenic and genetic analyses of seven H5N1 viruses isolated during these outbreaks were carried out. Antigenic analysis confirmed 64 to 256-fold reduction in cross reactivity compared with clade 2.2 viruses. The intravenous pathogenicity index of the isolates ranged from 2.80–2.95 indicating high pathogenicity to chickens. Sequencing of all the eight gene-segments of seven H5N1 viruses isolated in these outbreaks was carried out. The predicted amino acid sequence analysis revealed high pathogenicity to chickens and susceptibility to the antivirals, amantadine and oseltamivir. Phylogenetic analyses indicated that these viruses belong to clade 2.3.2.1 and were distinct to the clade 2.3.2.1 viruses isolated in Nepal. Identification of new clade 2.3.2 H5N1 viruses in South Asia is reminiscent of the introduction of clade 2.2 viruses in this region in 2006/7. It is now important to monitor whether the clade 2.3.2.1 is replacing clade 2.2 in this region or co-circulating with it. Continued co-circulation of various subclades of the H5N1 virus which are more adapted to land based poultry in a highly populated region such as South Asia increases the risk of evolution of pandemic H5N1 strains.

## Introduction

Since its first detection in 1996, highly pathogenic avian influenza (HPAI) H5N1 virus has become endemic in poultry in Southern People's Republic of China and parts of Southeast Asia [1]. Subsequently, the virus spread to over 60 countries in Asia, Europe and Africa infecting wild birds or domestic poultry with sporadic zoonotic transmission to humans and raised pandemic concern [2], [3]. During the last 15 years of circulation in poultry, the H5N1 virus has undergone significant genetic diversification and antigenic drift and 10 distinct virus clades (Clade 0 to Clade 9) with subclades have been reported [4]. The clade 2.2 H5N1 virus that caused widespread outbreaks in wild birds of Qinghai Lake in China subsequently spread westwards to the middle east and south Asia, Europe and Africa in 2006–2007 and got established in the poultry populations of some countries of Asia and Africa [5]. During this period the dominant virus clade in south-east Asia was clade 2.3.4. Recently, clade 2.3.2 viruses have been repeatedly detected in wild birds in Hong Kong, Japan, Russia and Mongolia and it was suggested that this clade may be established in migrating birds [6]. More recently, clade 2.3.2 has been repeatedly detected in wild birds in Europe and there has been an increased prevalence of this virus clade in poultry outbreaks in South East Asia [7], [8].

In South Asia, H5N1 virus was first detected in domestic poultry in India and Pakistan during February 2006 subsequently confirmed in Bangladesh, Nepal and Bhutan in March 2007, January 2009 and February 2010, respectively [9]. Although no human cases have been reported in India, the virus has infected 7 humans in Bangladesh, Myanmar and Pakistan with 1 death in Pakistan (http://www.who.int/influenza/human_animal_interface/EN_GIP_20111010CumulativeNumberH5N1cases.pdf accessed on 17.10.2011). India and Bangladesh are experiencing outbreaks of H5N1 virus every year since their first detection in 2006 and 2007 respectively [9], [10]. All the H5N1 viruses isolated from poultry and humans in South Asia until 2010 belonged to clade 2.2 [10], [11], [12], [13]. The first introduction of clade 2.3.2 H5N1 virus to South Asia was reported in Nepal in February, 2010 [8], [14]. Outbreaks in Eastern India and Bangladesh during the same period were due to clade 2.2 H5N1 viruses [13]. Here we report the first detection and the genetic and antigenic characterization of clade 2.3.2 H5N1 viruses in Indian poultry.

## Results and Discussion

The H5 virus infection was confirmed by RT-PCR, Real time RT-PCR in two out of seven duck carcass samples and the two allantoic fluids from the State Duck Breeding Farm, R.K. Nagar, and three chicken carcasses and two pooled swab samples from State Poultry Farm, Gandhigram of Tripura State in India. The neuraminidase (NA) subtype was confirmed to be N1 by one-step RT-PCR. The H5N1 viruses were isolated in special pathogen free (SPF) embryonated chicken eggs from all the positive samples. The viruses isolated included A/duck/India/02AF1/2011, A/duck/India/02CA10/2011, A/chicken/India/CL03485/2011; A/chicken/India/TR0383/2011; A/chicken/India/0301/2011; A/chicken/India/CA0302/2011; A/chicken/India/CA0303/2011. The results were communicated to the Department of Animal Husbandry, Dairying and Fisheries, Ministry of Agriculture, Government of India for initiation of control measures.

**Figure 1:**
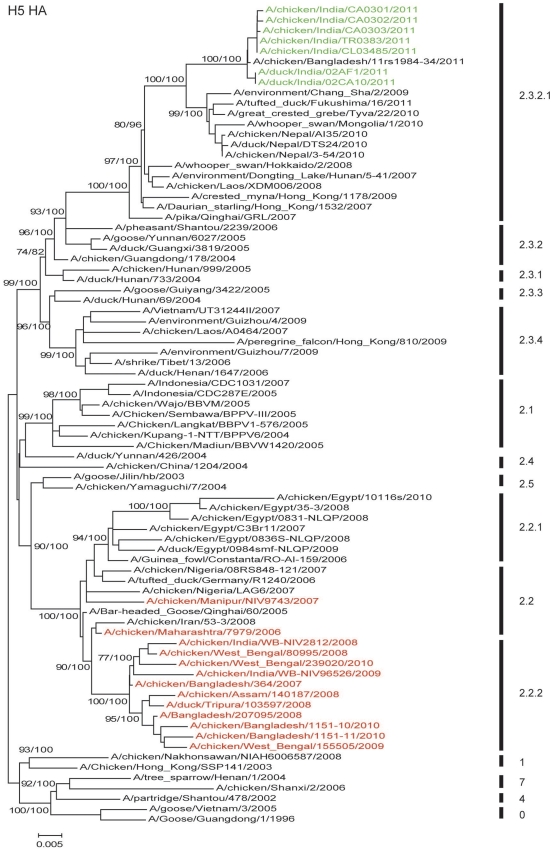
Phylogenetic relationships of the coding sequences of hemagglutinin (HA) genes of representative influenza A viruses. Analysis was based on full length or near full length sequences. The numbers next to the branch nodes indicate bootstrap values/posterior probabilities expressed as percentages from, respectively, 500 bootstrap replicates of a maximum likelihood tree and posterior probabilities from a MrBayes 3.2 analysis (see methods). Not all support valuess are shown due to space constraints. Numbers labeled on the HA tree refer to the WHO H5N1 clade designations (http://www.who.int/csr/disease/avian_influenza/guidelines/nomenclature/en). Viruses isolated in this work are in green and other recent Indian, Bangladesh and Bhutan viruses are in red. Scale bar, indicates nucleotide substitutions per site.

**Figure 2:**
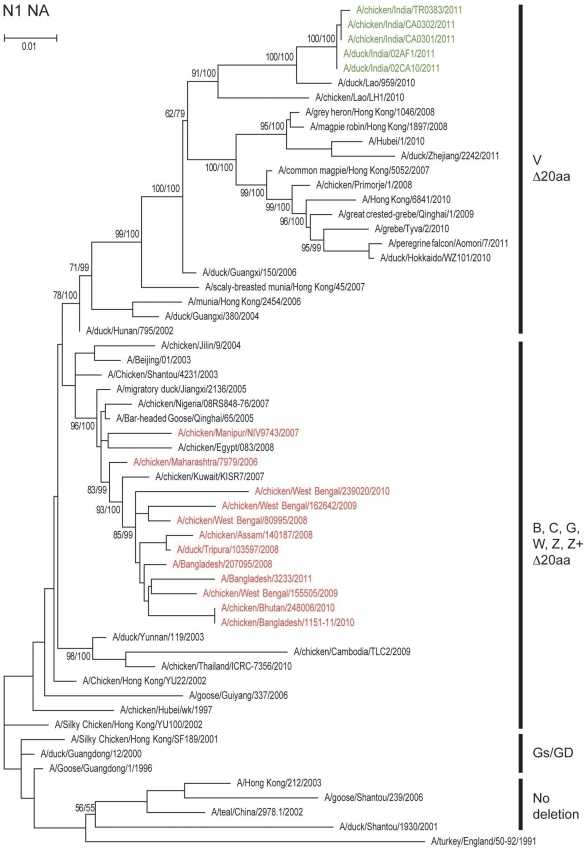
Phylogenetic relationships of neuraminidase (NA) genes of representative influenza A viruses. Details are as in the legend to Figure 1. The presence of deletions in the stalk region are indicated by Δ20aa. Genotype designations are from references 14 and 15.

**Figure 3:**
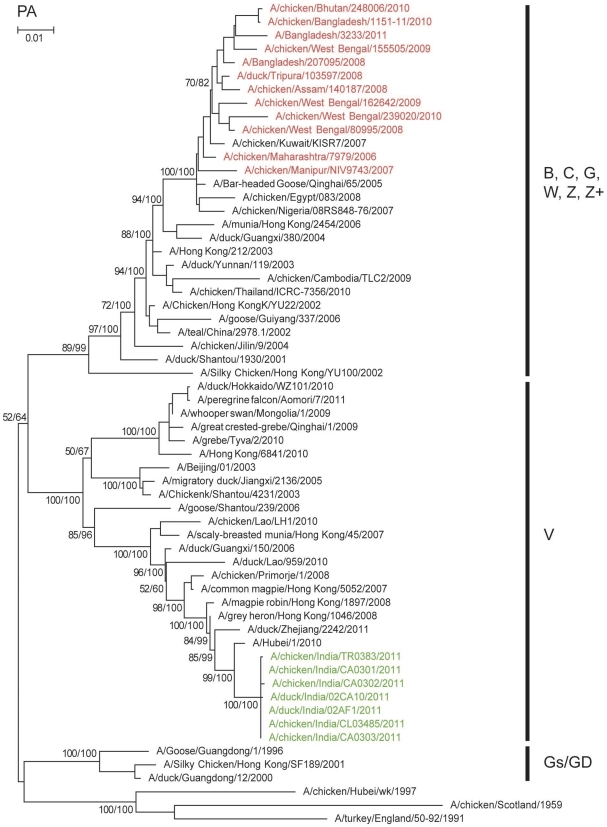
Phylogenetic relationships of polymerase acidic (PA) genes of representative influenza A viruses. Details are as in the legends to Figures 1 and 2.

Phylogenetic analysis of the HA genes (Figure 1) showed that the chicken and duck isolates of 2011 clustered with clade 2.3.2 viruses rather than with the clade 2.2 viruses reported earlier in India. Phylogenetically the 2011 Tripura isolates from the two farms clustered tightly together with 100% bootstrap value indicating a single introduction event. While they clustered within clade 2.3.2.1 with contemporary isolates from China, Vietnam, Hong Kong SAR, Japan, Mongolia, Nepal and Russia, the 2011 Tripura isolates were clearly distinct from these other viruses including those detected in Nepal (Figure 1). The Nepal isolates of 2010 shared only 97.6% similarity with 2011 Tripura isolates, and were phylogenetically much closer to those in Qinghai Lake and Mongolia in 2009. The phylogenetic analysis of the other seven genes shows similar evolutionary relationships to clade 2.3.2 viruses. The NA (Figure 2) and internal genes of the Indian isolates grouped with the recently isolated clade 2.3.2 viruses in genotype V based on classification described in [15] and [16] and were separate from the Indian and Bangladesh clade 2.2 viruses. The PA gene of the Indian isolates of 2011 formed a well supported group distinguishing genotype V and other clade 2.3.2 viruses from other subclades (Figure 3).

Antigenic analysis revealed 64 to 256-fold reductions in the HI titres in the seven isolates compared to clade 2.2 viruses isolated from this region in 2008 (Table 1). The results indicated that the 2011 Tripura clade 2.3.2 isolates were antigenically different from those clade 2.2 viruses isolated in 2008 from Tripura/West Bengal. The estimated percent divergence of HA amino acid sequences was 7.2 to 7.8 between clade 2.2 and clade 2.3.2 viruses and 0.0 to 0.5 within clade 2.3.2 viruses correlate with the difference in the antigenic relationship observed in cross neutralization HI titres.

**Table 1:** Antigenic characterization of Indian clade 2.3.2 H5N1 viruses.

Virus antigen	Virus clade	Hyper-immune chicken antiserum to		
		A/chicken/West Bengal/80995/2008(Clade 2.2)	A/duck/Tripura/103597/2008(Clade 2.2)	A/chicken/Tripura/CL03488/2011(Clade 2.3.2)
A/chicken/West Bengal/80995/2008	2.2	4096*	1024	32
A/duck/Tripura/103597/2008	2.2	4096	2048	64
A/duck/India/02AF1/2011	2.3.2	64	16	2048
A/duck/India/02CA10/2011	2.3.2	32	8	2048
A/chicken/India/CL03485/2011	2.3.2	64	16	1024
A/chicken/India/CA0302/2011	2.3.2	64	16	2048
A/chicken/India/0301/2011	2.3.2	64	8	1024
A/chicken/India/TR0383/2011	2.3.2	64	16	1024
A/chicken/India/CA0303/2011	2.3.2	64	16	2048
A/chicken/Tripura/CL03488/2011	2.3.2	64	16	2048

*- reciprocal of antibody titres by hemagglutination inhibition test.

The 2011 Tripura isolates have a multiple basic amino acids motif of PQRERRRKR/GLF in the HA cleavage region, which is identical to other clade 2.3.2 viruses, whereas the majority of clade 2.2 viruses have PQGERRRKKR/GLF. The receptor binding pocket of the HA retain amino acids Q-222 and G-224 (H5 numbering) that indicate preferential binding to avian-specific α-2, 3 sialic acid receptors [1].

*N-* linked glycosylation masks the oligosaccharides on the hemagglutinin (HA) and neuraminidase of influenza A virus from recognition by lectins of the innate immune system and thereby inhibit antibody mediated neutralization. Site specific glycosylation in the HA gene affects cleavage of the HA and thus the virulence of the influenza viruses [6]. Seven potential glycosylation sites (N-X-S/T where X is any amino acid except Proline) were present in the HA protein of the 2011 Tripura viruses at positions ^11^NST^13^, ^23^NVT^25^, ^140^NSS^142^, ^165^NNT^167^, ^286^NSS^288^, ^484^NGT^486^ and ^543^NGS^545^. There was one additional glycosylation site compared with Indian clade 2.2 viruses due to R140N mutation. This additional glycosylation site might indicate increased adaptation of the virus in land based poultry compared to clade 2.2 viruses [17] with no significant effect on the virulence of the virus as it is not located on the HA globular head [18]. The S129L substitution which is characteristic of clade 2.3.2 viruses [19] is present in all 2011 Tripura isolates. The amino acid mutations V223I and M230I in receptor binding domain of clade 2.2 viruses isolated from the largest human cluster in Egypt (Gharbya cluster) and the two human cases of Bangladesh are present in 2011 Tripura clade 2.3.2 isolates (http://www.recombinomics.com/News/03161103/H5N1_Dhaka_Cluster.html). Acquiring of these amino acid markers are significant since they are absent in Indian clade 2.2 viruses and are dominant in seasonal H1N1, H3N2 and influenza B viruses. The acquisition of additional glycosylation site and accumulation of these mutations might indicate that the H5N1 viruses are evolving in land based poultry and might acquire the ability to support human transmission [17]. Hence, there is a need to monitor the evolution of these clade 2.3.2.1 viruses.

Changes in the amino acids affecting the receptor biding capacity had been predicted to influence the detection limit of HI test using chicken RBCs [20]. The K189R and S129L HA mutations which are predicted to increase the binding affinity for SA*α*2,3Gal [21] and is present in all the 2011 Tripura isolates. The K189R mutation is also present in the Indian clade 2.2 virus A/chicken/India/80995/2008 which was used for antigenic characterization. However, there was no significant difference in the cross reactivity compared with the other clade 2.2 virus which did not possess this mutation (A/duck/Tripura/103597/2008) (Table 1). Hence, though this mutation might increase the receptor binding capacity of the virus [21], its presence may not affect the cross antigenic reactivity between clade 2.2 and clade 2.3.2 viruses.

In common with other clade 2.3.2 and 2.2 viruses, the neuraminidase has a 20 amino acid deletion in the stalk of the protein. The predicted amino acid sequence analysis of the NA and matrix genes revealed that the virus isolates are likely to be sensitive to commonly used influenza drugs such as amantadine and oseltamivir. The E627K mutation in the PB2 protein, associated with increased virulence of influenza A H5N1 viruses in mammals was not present in the 2011 Tripura isolates from India. Deletion of five amino acids in the N-terminal of the NS1 protein from position 80 to 84 (corresponding to nucleotides 263–277) which might enhance cytokine expression by macrophages [22] is present in 2011 Tripura clade 2.3.2 viruses similar to other Indian clade 2.2 viruses. Molecular markers for possible increase in the virulence of this virus in chicken and mice and also inhibition of host immune responses viz. S42 [23], A144 [24] and C-terminal amino acid motif of ESKV [25] in the NS 1 protein are present in 2011 Tripura isolates and in South Asian clade 2.2 viruses. The pathogenicity of the 2011 Tripura viruses in mice needs to be ascertained to assess their possible public health implications.

The intravenous pathogenicity index calculated for all the seven isolates ranged from 2.80–2.95 indicating high pathogenicity to chickens. However, the 61% mortality observed in the outbreak at State Duck Breeding Farm was unusually high for duck species. Further investigations are needed to ascertain whether the presence of concurrent infection with other infectious agents such as duck viral enteritis or duck viral hepatitis contributed to the increased mortality. In chickens, even though 100% mortality was recorded in one shed, no mortality was observed in other sheds indicating that strict biosecurity measures prevented onward spread.

Interestingly, no mortality was observed in other poultry within 3 KM radius of the first outbreak in the one month that separated the two outbreaks and all the samples collected during the intensive post-outbreak surveillance program were negative for avian influenza (data not shown). Hence, there is a possibility that the introduction to the Gandhigram farm was not through direct transmission from the index farm. The role of fomites or local migratory birds needs to be considered.

It was reported that clade 2.3.2 viruses were circulating in Bangladesh during January to February, 2011 [26] which coincides with the time of Tripura outbreak in India. WHO in its recent analysis of antigenic and genetic characteristics of zoonotic influenza viruses and development of candidate vaccine viruses for pandemic preparedness (http://www.who.int/influenza/resources/documents/2011_09_h5_h9_vaccinevirusupdate.pdf accessed on 10th October, 2011) reported that the 2011 Tripura viruses group with 2011 Bangladesh and 2010 and 2011 Myanmar clade 2.3.2.1 viruses. Based on the phylogenetic relationship published by the WHO, it can be concluded that the clade 2.3.2.1 viruses had circulated in Myanmar and spread to Bangladesh and India. The most probable route of this transmission could be the movement of land based poultry or local migratory birds.

**Figure 4:**
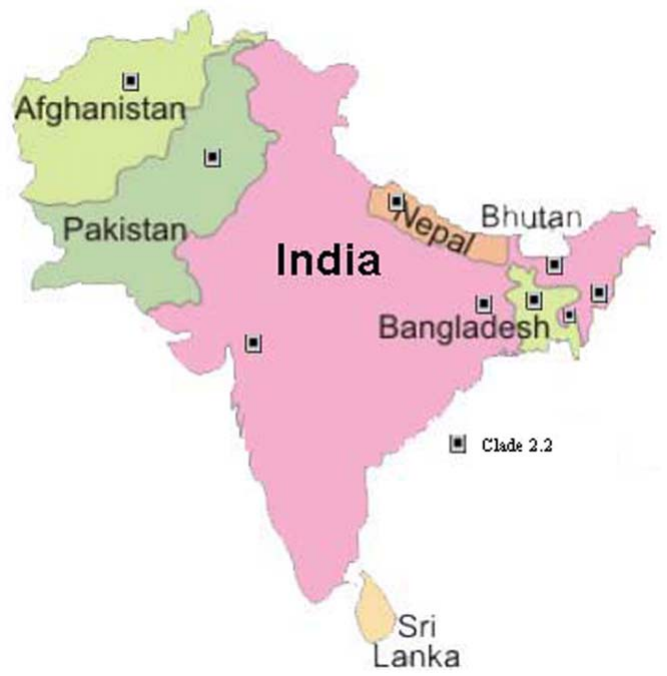
Circulation of H5N1 viruses in South Asia till 2009.

**Figure 5:**
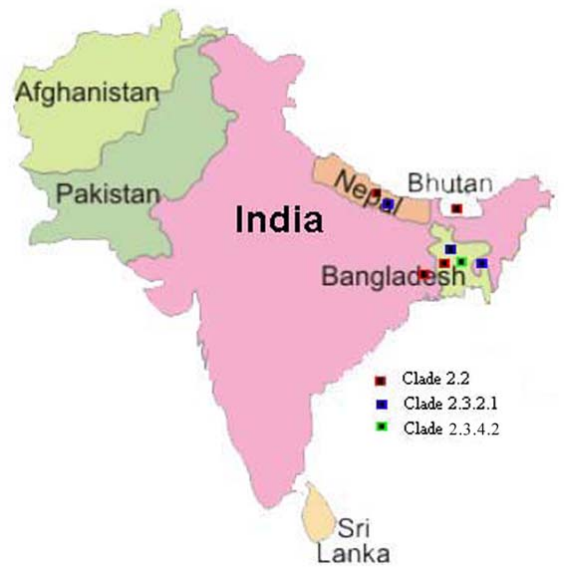
Circulation of H5N1 viruses in South Asia since 2010.

Following the repeated detection H5N1 clade 2.3.2 viruses in wild birds in Hong Kong in 2006–2008 and in waterfowl and poultry in Russia and Japan, we suggested that this virus clade may have become endemic in wild birds and may be spreading via long distance bird migration [19]. In 2009/10, this virus clade has been repeatedly detected in China, Japan, Mongolia and Russia [5], [27]. However in South Asia, till 2009, only clade 2.2 H5N1 viruses were reported (Figure 4). In early 2010, clade 2.3.2 was confirmed in Nepal (the first reported introduction of this virus into South Asia) [8], [14] and in poultry and wild birds in Romania and Bulgaria, respectively in March 2010 [8]. The possible dissemination of influenza A H5N1 throughout Eurasia through wild migratory birds has been previously discussed [19], [28]. The emergence and spread of clade 2.3.2.1 of HPAI H5N1 virus in South Asian region (Figure 5) supports the contention that this virus clade is probably established in wild birds and land based poultry and is spreading its geographical range just as clade 2.2 before it. The identification of new clade 2.3.2.1 H5N1 viruses in South Asia (India, Bangladesh and Nepal) that are phylogenetically closely related to those isolated in Qinghai Lake, China and Mongolia in 2009 and 2010 is reminiscent of the introduction of clade 2.2 viruses in this region in 2006/7 [28]. It is now important to monitor whether the clade 2.3.2.1 is replacing the previous clade 2.2 in this region or co-circulating with it. The WHO report on antigenic and genetic characteristics of zoonotic influenza viruses and development of candidate vaccine viruses for pandemic preparedness (http://www.who.int/influenza/resources/documents/2011_09_h5_h9_vaccinevirusupdate.pdf) also reveals isolation of clade 2.3.4.2 viruses in Myanmar and Bangladesh which showed reduced reactivity with post-infection ferret antisera against the clade 2.3.4 viruses. Continued circulation of the H5N1 viruses of various subclades which are more adapted to land based poultry in a highly populated region such as South Asia might lead to evolution of pandemic strains with devastating consequences. Hence, there is an urgent need for faster sharing of data in public domain or through bilateral/international agencies for better management of control of the virus spread and its evolution in South Asia.

## Materials and Methods

### Clinical samples

On 3^rd^ February, 2011 mortality was observed in parent duck flocks of *Khaki campbell* (*Anas platyrhynchos*) breed in State Duck Breeding Farm, R.K. Nagar, Agartala District of Tripura State and continued till 17^th^ February, 2011 until culling. During this outbreak, 1796 (863 adult and 933 ducklings) out of the total 2948 (1448 adults and 1500 ducklings) birds died. After initial testing at Eastern Regional Disease Diagnostic Laboratory (Kolkata, West Bengal), 7 dead birds (4 adult ducks and 3 ducklings) and 2 infected allantoic fluid samples were received at High Security Animal Disease Laboratory (HSADL), Bhopal for confirmation. Field visit by HSADL scientist during February 19–20 revealed no indication of morbidity/mortality in poultry beyond the 3 Km radius of the outbreak epicenter and no unusual morbidity and mortality was reported by the farmers for the next one month. On 5^th^ March, 2011 a second outbreak was reported in *Croiler* breed of chickens housed at State Poultry Farm, Gandhigram, in the same District located approximately 10 Km North West from the previous outbreak. All 380 chickens in one of the sheds died (mortality 100%) and the remaining 13,936 birds within the 3 KM radius of the outbreak were culled. A total of 3 chicken carcass and 8 swab (4 cloacal and 4 tracheal) samples were received at HSADL on 6^th^ March, 2011.

### Identification by RT-PCR and real time RT-PCR

Viral RNA was extracted from the clinical samples using QIAamp Viral RNA Mini Kit (Qiagen, Germany) as recommended by the manufacturer. One step RT-PCR and real time RT-PCR for identification of Type A influenza virus and HA subtype was carried out as previously described [29], [30]. The NA subtyping RT-PCR was carried out as previously described [31].

### Virus isolation

Dead carcasses were autopsied and organs were collected and pooled for each bird. Cloacal and tracheal swabs were pooled separately and processed for virus isolation. Virus isolation was carried out in 9–11 day old embryonated specific pathogen free (SPF) chicken eggs as previously described [32].

### Antigenic characterization

Antigenic relationship between clade 2.3.2 and clade 2.2 H5N1 influenza viruses isolated from India in 2008 was tested by haemagglutination inhibition (HI) test using polyclonal chicken antisera raised against two clade 2.2 viruses and one clade 2.3.2 virus [5].

### Intravenous pathogenicity index

Intravenous pathogenicity index (IVPI) was carried out as per the standard protocol recommended by World Health Organization [33]. The animal experiments were carried out at the BSL-4 containment animal wing of HSADL, IVRI, Bhopal as per the guidelines of Institutional Animal Ethics Committee and Committee for the Purpose of Supervision and Control of Experiments on Animals (CPCSEA), Ministry of Environment and Forests, Govt. of India (Approval no. 42/IAEC/HSADL/09 dated 08.07.2010). Briefly, the 1∶10 diluted allantoic fluid of A/chicken/India/CL03485/2011 isolate with a HA titer of 1∶32 was inoculated intravenously into eight 5 weeks old avian influenza virus (AIV) antibody negative chickens and scored based on the symptoms/death of the birds. Eight control chickens were inoculated with 1×PBS.

### PCR amplification and sequencing

Sequencing of complete genome (all 8 segments) of 7 H5N1 viruses isolated from various sample types in both the outbreaks (one viral allantoic fluid of duck origin, 1 virus from duck carcass, 3 from chicken carcass and 1 each from cloacal and tracheal swab pools) was carried out. RT-PCR for amplification of various gene fragments with overlapping segment-specific primers was carried out using Platinum Taq High Fidelity (Invitrogen, USA) as described previously [12]. The PCR products were gel purified using QIAquick gel extraction kit (Qiagen, Germany) and sequenced using specific PCR primers with BigDye® Terminator v3.1 Cycle Sequencing Kit (Cat. 4337455, Applied Biosystems, USA) in 3130- Genetic Analyzer (Applied Biosystems, USA). Nucleotide sequences reported in this study have been submitted to the GenBank (accession numbers CY089410-CY089425, CY089468- CY089477, CY092115- CY092120, CY092121- CY092132, CY092133- CY092144, CY089468- CY089477).

### Analysis of genetic data

Maximum likelihood trees with 500 bootstrap replicates were inferred using MEGA5 [34] based on the GTR+I+G model suggested by jModelTest [35]. Confirmation of the trees was provided by a Bayesian analysis in MrBayes3.2 [36] run for 5,000,000 generations and sampled every 1,000 generations.
